# Inadvertent Lead Placement In The Left Ventricle: A Case Report And Brief Review

**Published:** 2009-07-01

**Authors:** David D McManus, Mary-Lee Mattei, Karen Rose, Jason Rashkin, Lawrence S Rosenthal

**Affiliations:** Electrophysiology Section, Division of Cardiology, Department of Medicine, University of Massachusetts Medical Center, Worcester, MA 01655

**Keywords:** left ventricular pacing, complication of pacemaker or defibrillator implantation, lead extraction, left ventricular lead

## Abstract

Inadvertent lead placement in the left ventricle (LV) is an uncommon and often under-diagnosed complication of cardiac device implantation. Thromboembolic (TE) events are common and usually secondary to fibrosis or thrombus formation on or around the lead. Anticoagulation can prevent TE events. Percutaneous and surgical LV lead extractions have been performed successfully, but the risks of percutaneous lead removal are not well-defined. In this report, we describe a case of inadvertent LV lead placement and briefly review the contemporary literature.

## Case Report

A 78 year-old man with a history of paroxysmal atrial fibrillation (AF) presented with syncope and underwent implantation of a dual-chamber Medtronic KDR703 pacemaker while in AF. A Medtronic 4068 ventricular lead was implanted and found to have a threshold of 1.2 Volts (V) at 0.5 ms, a current of 1.8 milliamps and an impedance of 887 ohms. R-wave sensing was noted to be 16.6 mV. A Medtronic 4568 atrial lead was implanted but threshold testing was not performed and sensing was not reported. The pacemaker was programmed to VVIR 60-120.

Two months later, the patient developed left upper extremity weakness and a computed-tomography (CT) scan confirmed the clinical diagnosis of a right parietal cerebrovascular accident (CVA). The CVA was attributed to thromboembolism secondary to AF and the patient was initiated on warfarin therapy with a target INR of 2.0-3.0.

**Figure 1:**
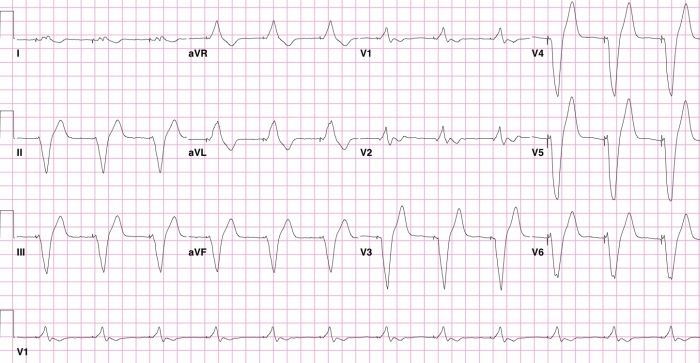
12-lead ECG obtained from a patient with an endocardial LV lead with a RBBB-configuration of the stimulated QRS complex

**Figure 2:**
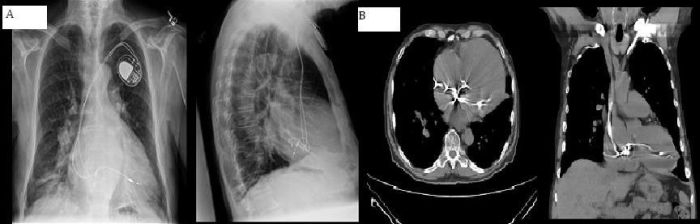
A. PA and lateral chest radiograph from a patient with inadvertent LV lead placement. The lead in the LV is clearly seen posteriorly on the lateral view. B. Chest CT from the same patient. The ventricular lead is seen passing from the RA to the LA through an ASD and from the LA to the LV through the mitral valve.

The patient was referred to our clinic after his ventricular lead was noted to have low impedance and a decrease in sensed R wave amplitude on routine interrogation. The patient's ECG is shown (Figure 1). The diagnosis of inappropriate lead placement in the LV was suspected on the basis of the paced right bundle branch block (RBBB) pattern noted on this ECG. The diagnosis was confirmed by anterior and lateral chest radiography (Figure 2). A non-contrast chest CT scan shows the ventricular lead coursing from the right atrium into the left atrium (LA) through an atrial septum defect (ASD) (Figure 2).

Device interrogation revealed a ventricular lead threshold of 1.25 V at 0.4 ms, R wave sensing of 2.8-4.0 mV and a lead impedence of 248 ohms. The patient was in AF during interrogation with a slow, irregular ventricular response. No atrial capture could be demonstrated and no P waves were sensed.

Due to suspected ventricular lead dysfunction and concern that sudden loss of ventricular pacing would lead to a recurrence of syncope, the patient underwent lead revision. A St. Jude 1688 lead was inserted into the right ventricle under fluoroscopic guidance using anterior and lateral views and the existing atrial lead and LV lead were capped. The patient's warfarin dose was increased to achieve a target INR of 2.5-3.5. The patient was discharged home without event. At the time of this report submission, the patient has had no further TE complications. Given the patient's age and comorbidities, should he experience further TE complications despite appropriate anticoagulation or if he develops an absolute contraindication to warfarin, we would consider percutaneous LV lead extraction with on-site cardiothoracic surgery back-up.

## Discussion

### Review

Inadvertent LV pacing due to malpositioned endocardial pacing leads is a known, but uncommon complication of permanent pacemaker implantation. A Medline search of English-language articles revealed over 40 cases of inadvertent LV lead placement. As in our case, passage of the lead through an ASD or patent foramen ovale was the most common reported cause of inadvertent LV pacing [1,2]. Inadvertent LV pacing has also been described in the setting of ventricular septal and apical perforation  [3]. Erroneous cannulation of the subclavian artery and retrograde passage of the lead into the LV across the aortic valve has also been reported [4].

TE complications occurred in approximately forty percent of patients after inadvertent LV lead placement  [3,5-7]. Thromboembolism is thought to have resulted from thrombus formation and fibrosis on or around the site of lead implantation. Case reports describe syncope, amaurosis fugax, aphasia and hemiplegia as the most common presenting complaints of patients with thromboembolism  [3,6,8]. TE events have been reported as soon as one day after lead implantation and up to several years later  [3]. Thromboembolism has been described in several patients receiving antiplatelet therapy  [7]. Other significant complications of inadvertent LV lead placement included endocarditis, pericardial effusion, vascular damage and peripheral arterial thrombosis   [7].

Time from implantation to diagnosis is highly variable and appears to influence treatment strategy. Percutaneous LV lead removal has been successful but only in patients diagnosed less than one year after LV lead placement  [4,9,10]. Fifty percent of patients with and twenty-five percent of patients without TE complications underwent surgical lead removal. Inspection of explanted leads showed adherent thrombus in several cases, including those explanted from asymptomatic patients and patients receiving aspirin. Notably, transthoracic and transesophageal echocardiography failed to identify thrombus preoperatively in several of these cases [7].

Of patients with inadvertent LV lead placement treated without surgery, only one case of anticoagulation failure with warfarin was reported. This event occurred in the setting of a subtherapeutic INR (1.6)  [3]. Data from 20 patients with intentional endocardial LV lead placement for cardiac resynchronization indications were also reviewed for TE complications  [11,12]. These data also show a low incidence (one patient) of thromboembolism with anticoagulation.

### Diagnosis

Early activation of the LV during pacing creates a RBBB-pattern on ECG and, as in our case, is often the key to diagnosis of inadvertent LV lead placement (Figure 1). This ECG pattern has good sensitivity but inadequate specificity for LV lead malposition since a similar pattern may be seen in the setting of RV dilatation, septal pacing or coronary sinus pacing  [13]. Most patients with malpositioned LV leads, as in our case, have normal pacing thresholds and this cannot be recommended as a screening tool. As the patient had normal lead impedances on implantation, we do not hypothesize that the low impedances noted in our case relate directly to endocardial LV lead placement. Chest radiography, in contrast, is often helpful in delineating the position of the ventricular lead and can therefore be used a confirmatory test in suspected lead malposition (Figure 2).

It is advisable that all patients undergoing device implantation have a 12-lead ECG recorded during pacing. Consideration should also be given to routine post-implantation anterior and lateral chest radiography. At a minimum, those with a RBBB-pattern during pacing should receive both an anterior and lateral chest radiograph. If lead malposition is suspected, echocardiography can be helpful in confirming the diagnosis and determining the course of the lead but, due to its poor sensitivity in published reports, should not be used to 'rule out' the presence of thrombus  [4,14].

### Management

If a diagnosis of inadvertent lead placement in the LV is made immediately after implant, percutaneous lead extraction can reduce the risk of future TE events without the need for lifelong anticoagulation. While percutaneous LV lead extraction has been performed successfully up to 9 months after implantation, this procedure carries a risk of systemic embolization from lead manipulation, especially if a laser sheath is used  [9,10]. While thrombus formation has occurred in the first 24-48 hours after LV lead implantation, percutaneous lead extraction is appealing in this period because it does not require the use of a laser sheath and thus may have lower procedural risk than surgical lead removal with use of cardiopulmonary bypass.

For those patients in whom the diagnosis of inadvertent LV lead placement is delayed, anticoagulation with warfarin is reasonable as no TE events have been reported in patients with an INR of 2.5-3.5  [12]. Use of antiplatelet therapy as a sole means of TE prophylaxis in patients with inadvertent LV lead placement is not advisable. Surgical LV lead extraction also warrants consideration, especially if cardiac surgery has to be performed for another indication. Surgical lead extraction may be preferable in younger patients in whom the risk of surgery is lower and the planned duration of warfarin longer.

### Conclusions

While uncommon, inadvertent LV lead placement is a potentially devastating complication of pacemaker implantation. Appropriate analysis of the paced QRS pattern on ECG and post-implantation chest radiograph may reduce morbidity and mortality by promoting early recognition and treatment. Percutaneous lead extraction is an option for patients diagnosed in the immediate post-operative period. Surgical lead removal or lifelong anticoagulation should be considered for patients in whom the diagnosis of inadvertent LV lead placement is delayed.

## References

[R1] Engstrom A (2006). Inadvertent malposition of a transvenous pacing lead in the left ventricle. Herzschrittmacherther Elektrophysiol.

[R2] Lee WL (1995). Transvenous permanent left ventricular pacing. Angiology.

[R3] Sharifi M (1995). Inadvertent malposition of a transvenous-inserted pacing lead in the left ventricular chamber. Am J Cardiol.

[R4] Reising S (2007). A stroke of bad luck: left ventricular pacemaker malposition. J Am Soc Echocardiogr.

[R5] Ghani M (1993). Malposition of transvenous pacing lead in the left ventricle. Pacing Clin Electrophysiol.

[R6] Sharifi M (1994). Left heart pacing and cardioembolic stroke. Pacing Clin Electrophysiol.

[R7] Van Gelder BM (2000). Diagnosis and management of inadvertently placed pacing and ICD leads in the left ventricle: a multicenter experience and review of the literature. Pacing Clin Electrophysiol.

[R8] Read PA (2005). Ventricular tachycardia and amaurosis fugax following inadvertent left ventricular pacing. Int J Cardiol.

[R9] de Cock CC (2003). Successful percutaneous extraction of an inadvertently placed left ventricular pacing lead. Europace.

[R10] Trohman RG (1991). Successful percutaneous extraction of a chronic left ventricular pacing lead. Pacing Clin Electrophysiol.

[R11] Jais P (1998). Endocardial biventricular pacing. Pacing Clin Electrophysiol.

[R12] van Gelder BM (2007). Transseptal endocardial left ventricular pacing: an alternative technique for coronary sinus lead placement in cardiac resynchronization therapy. Heart Rhythm.

[R13] Yang YN (2003). Safe right bundle branch block pattern during permanent right ventricular pacing. J Electrocardiol.

[R14] Vanhercke D (2008). Eight years of left ventricle pacing due to inadvertent malposition of a transvenous pacemaker lead in the left ventricle. Eur J Echocardiogr.

[R15] Pasquie JL (2007). Long-term follow-up of biventricular pacing using a totally endocardial approach in patients with end-stage cardiac failure. Pacing Clin Electrophysiol.

